# Cancer-associated fibroblasts impact the clinical outcome and treatment response in colorectal cancer via immune system modulation: a comprehensive genome-wide analysis

**DOI:** 10.1186/s10020-021-00402-3

**Published:** 2021-10-30

**Authors:** Yu-feng Chen, Zhao-liang Yu, Min-yi Lv, Ze-rong Cai, Yi-feng Zou, Ping Lan, Xiao-jian Wu, Feng Gao

**Affiliations:** 1 Department of Colorectal Surgery, The Sixth Affiliated Hospital, Sun Yat-Sen University, 26 Yuancun Erheng Rd, Guangzhou, 510655 Guangdong People’s Republic of China; 2 Guangdong Provincial Key Laboratory of Colorectal and Pelvic Floor Diseases, Guangdong Institute of Gastroenterology, The Sixth Affiliated Hospital, Sun Yat-Sen University, Guangzhou, Guangdong People’s Republic of China

**Keywords:** Fibroblast-related gene signature, Colorectal cancer, Prognosis, Chemotherapy

## Abstract

**Background:**

Cancer-associated fibroblasts (CAFs) in the tumour microenvironment are associated with poor prognosis and chemoresistance in multiple solid tumours. However, there is a lack of universal measures of CAFs in colorectal cancer (CRC). The aim of this study was to assess the utility of a fibroblast-related gene signature (FRGS) for predicting patient outcomes and reveal its relevant mechanism.

**Methods:**

The GSE39582 dataset, which includes 316 CRC patients who did not receive adjuvant chemotherapy was used as a discovery cohort to identify the prognostic fibroblast-related genes (FRGs). A total of 1352 CRC patients were divided into one training cohort (GSE39582, n = 461) and two validation cohorts (TCGA, n = 338; meta-validation, n = 553) for the construction of the FRGS and the verification of its prognostic value in stage II/III CRC patients. Functional annotation and analysis were performed to explore the underlying mechanism. The ability of the FRGS to predict immunotherapy response was further tested in a clear cell renal cell carcinoma (ccRCC) cohort.

**Results:**

An 11-gene signature that had prognostic value for stage II/III CRC patients in both validation cohorts was developed (TCGA cohort: HR = 1.90, 95% CI 1.16–3.12, *P* < 0.01; meta-validation cohort: HR = 1.95, 95% CI 1.39–2.73, *P* < 0.001). A high level of CAFs was correlated with worse prognosis in CRC patients who did not receive adjuvant chemotherapy (HR = 3.63, 95% CI 2.24–5.88, *P* < 0.001). Importantly, patients in the low-risk group were found to be benefit from chemotherapy (*P* < 0.01), but not in the high CAF group (*P* > 0.05). Similar results were found in the TCGA cohort. Integrated with clinical characteristics, the FRGS was confirmed to be an independent prognostic factor in the multivariate analysis after adjustment for tumour TNM stage (GSE39582 cohort: HR = 3.19, 95% CI 1.88–5.41, *P* < 0.001; TCGA cohort: HR = 5.00, 95% CI 1.58–15.85, *P* = 0.007; meta-validation cohort: HR = 2.99, 95% CI 1.44–6.21, *P* = 0.003). Furthermore, the enrichment analysis found that the antitumour immune response was suppressed and the infiltration of CD4 T cells and M1 macrophages was depressed in the high CAF group. The FRGS was also found to have value in predicting for immunotherapy response in the ccRCC cohort.

**Conclusions:**

The 11-gene FRGS had independent prognostic value for CRC patients, as well as utility in the prediction of benefit from chemotherapy. CAFs in the tumour microenvironment might have an impact on the prognosis of CRC patients via inhibiting immune response.

**Supplementary Information:**

The online version contains supplementary material available at 10.1186/s10020-021-00402-3.

## Introduction

Colorectal cancer (CRC) is the third most common and the second most lethal malignancy globally, representing approximately 10% of overall cancer cases and deaths (Sung et al. 2021). Its biological heterogeneity leads to diversity in the survival of CRC patients. Approximately 25% of patients with stage II and III CRC suffer from tumor recurrence despite receiving radical surgery and adjuvant chemotherapy. Moreover, the incidences of chemotherapy-associated death and significant side-effects are approximately 0.5–1% and 20%, respectively (Andre et al. 2009, 2015; Quasar Collaborative Group 2000; Quasar Collaborative Group et al. 2007). Therefore, a new strategy based on reliable markers to stratify subgroups with different risks of treatment response, tumour recurrence and tumour-specific death based on reliable markers is an urgent need for precise treatment of CRC patients.

Cancer-associated fibroblasts (CAFs) are considered to be one of the critical components of the tumour microenvironment (TME) in CRC because they provide physical support for epithelial cells and functioning as key regulators in tumorigenesis in a context-dependent manner (Kobayashi et al. 2019). The infiltration of CAFs has been reported to correlate with CRC patient prognosis and drug response. Previous studies have revealed that CAFs contribute to an immunosuppressive TME and promote tumour invasion and metastasis via the secretion of different kinds of cytokines and chemokines, such as IL-6 and CCL2 (Kalluri 2016). CAFs are also reported to be mediators of response to immune checkpoint inhibitors (Tauriello et al. 2018). Furthermore, emerging evidence has demonstrated that CAFs might confer substantial resistance to cancer therapeutics by impairing drug delivery and several biochemical signaling pathways (Kalluri 2016). Our previous study showed that CAFs could induce the chemoresistance of CRC through enhancement of the stemness of cancer cells (Tang et al. 2018). Additionally, our study showed that characteristics of the TME might be able to predict patient clinical outcome (Zou et al. 2019). The above studies imply that CRC patients might benefit from improved selection based on individual fibroblast-related characteristics. However, there is no clinical method of selecting patients with high levels of fibroblast-related risk factors.

Therefore, we analyzed the expression of fibroblast-related genes (FRGs) in CRC transcriptional data in this study, and combined multiple fibroblastic genes to construct a prognosis-related signature. Furthermore, the prognostic prediction value of this fibroblast-related gene signature (FRGS) was validated systematically. This study will aid in therapeutic decision making for CRC patients.

## Materials and methods

### Patient cohorts

Six public cohorts with gene expression data derived from fresh-frozen CRC samples were evaluated retrospectively, including The Cancer Genome Atlas (TCGA) CRC cohort and five datasets from the Gene Expression Omnibus (GEO) database (GSE39582, GSE14333, GSE17536, GSE37892 and GSE33113). The GSE39582 dataset including 309 CRC patients who did not receive adjuvant chemotherapy was used as the training cohort, while the TCGA CRC cohort and a meta-validation cohort containing the other four datasets were used for independent validation. The gene expression profiles of TCGA CRC cohort were obtained from Broad GDAC Firehose (http://gdac.broadinstitute.org/), as transcripts per million (TPM) values of level-three RNA-seq data on a log2 scale were used in our previous study (Zou et al. 2019). The other five datasets were directly obtained from the GEO database with the Bioconductor package ‘GEOquery’. A total of 1656 CRC patients were ultimately included in this study. The batch effects were corrected with the ‘combat’ algorithm implemented in the R package ‘sva’, and the z-scores for each gene were used during the following analyses. Data were collected from Sep 27 to Dec 26, 2018.

### Construction and validation of the CAF-related gene signature

To construct a prognostic fibroblast-related gene signature (FRGS), a list of fibroblast-related genes was first identified by including all gene sets using the keyword ‘fibroblast’ in MSigDB (version 6.2), and genes that were measured in each platform were selected. Prognostic genes were further selected using the log-rank test with 1000 randomizations (95% portion of samples each time) to assess the correlation between each gene and patients disease-free survival (DFS) in GSE39582 dataset. CRC patients in all stages were included in this stage. The genes that repeatedly showed significance were selected as the candidates for the FRGS. To minimize the risk of overfitting, a Cox proportional hazards regression model combined with the least absolute shrinkage and selection operator (LASSO) method (glmnet, version 2.0-16) was employed. The penalty parameter was estimated by tenfold cross-validation in the training dataset at one SE beyond the minimum partial likelihood deviance.

To separate patients into low- and high-risk groups, the optimal cut-off of each gene was determined with the time-dependent receiver operating characteristic (ROC) curve (survival ROC, version 1.0.3) for 5-year DFS in the training dataset. The Kaplan–Meier method was used to estimate survival. Then, the prognostic value of the FRGS was assessed in stage II/III CRC patients in the training and independent validation cohorts with univariate and multivariate analyses. A P value less than 0.05 was considered statistically significant.

### Functional annotation and analysis

To explore the biological characteristics of the FRGS, enrichment analysis of the genome-wide differentially expressed genes between different CAF risk groups was performed with the R package ‘gProfileR’ with the TCGA CRC dataset. To clarify the potential biological pathways, gene set enrichment analysis (GSEA) was conducted with the Bioconductor package ‘HTSanalyzeR’ (Wang et al. 2011). The Estimation of STromal and Immune cells in MAlignant Tumor tissues using Expression data (ESTIMATE) algorithm was further used to estimate the proportions of stromal and immune cells. The levels of different infiltrating immune cells, such as monocytes, lymphocytes and neutrophils, were calculated with CIBERSORT.

### Evaluation of the ability of the FRGS to predict the response to PD-1 treatment

To investigate the value of the FRGS for predicting the response to immune checkpoint inhibitors, a cohort with patients with advanced clear cell renal cell carcinoma who were mostly treated with PD-1 targeted treatment was used, and the patients were divided into a high-risk group and a low-risk group. The progression-free survival (PFS) and overall survival (OS) of the different groups were compared using the Kaplan–Meier method.

### Statistical analysis

Statistical analyses were performed with SPSS (version 22.0.0, IBM SPSS statistics, IBM Corporation, Armonk, NY) and R software (version 3.5.1; http://www.Rproject.org). Descriptive statistics, including means and standard deviations (SD) or medians and interquartile ranges (IQR) for continuous factors and frequencies for categorical factors, were computed for all variables. Continuous values were compared between different groups using Student’s t tests. Univariate analysis of the association of the FRGS and other clinicopathological factors with survival was performed using the log-rank test. Factors that were significantly associated with DFS and OS in univariate analyses were included in the Cox proportional hazards regression multivariate analysis. The C-index was calculated with the ‘survcomp’ package (version 1.32.0). A *P* value less than 0.05 was considered as statistically significant in all tests.

## Results

### Establishment and training of the FRGS in GSE39582 cohort

**Fig. 1:**
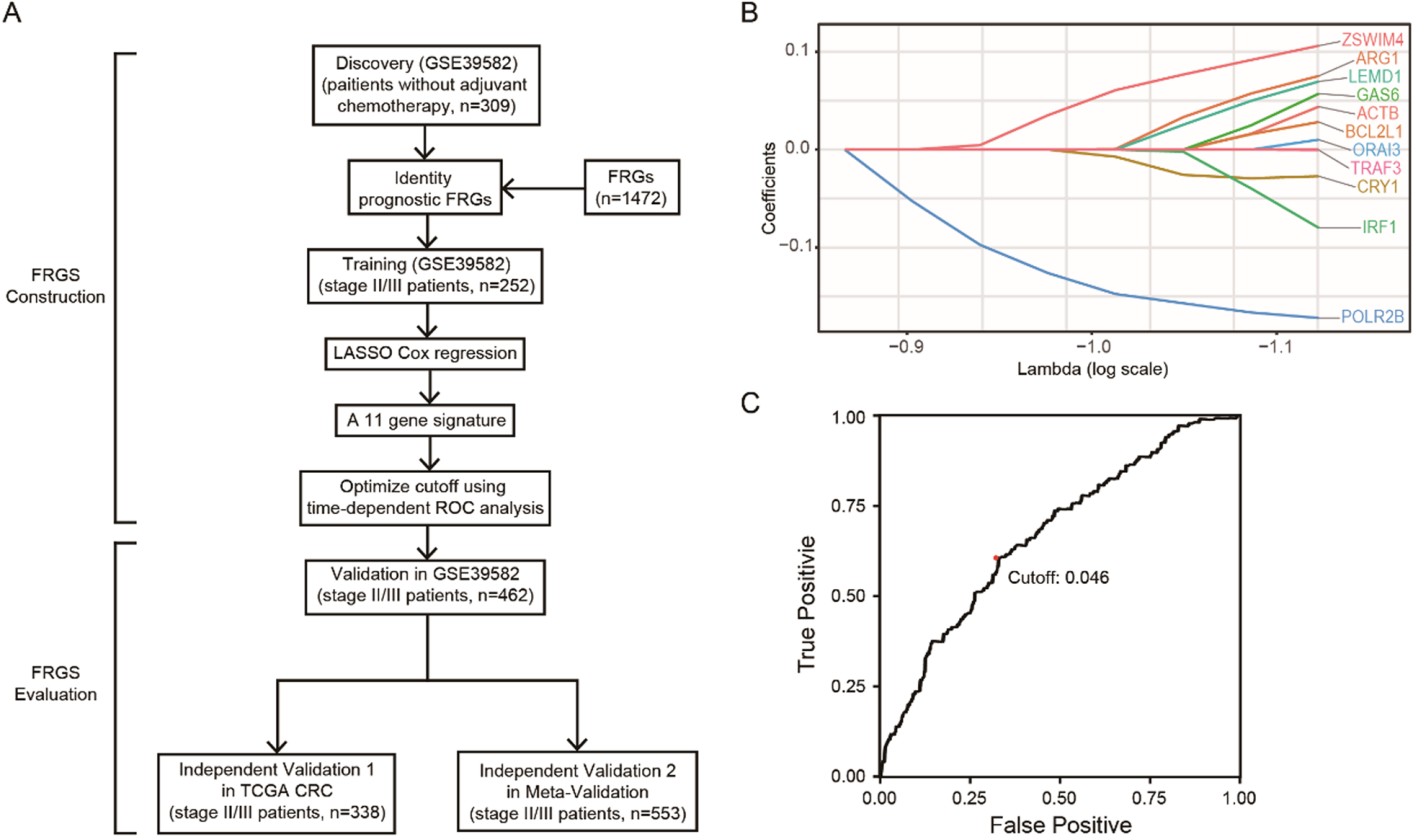
Establishment and verification of FRGS. **A** Schematic flow chart of study design. **B** A total of 11 fibroblast-related genes selected in LASSO Cox regression. **C** The optimal cut-off obtained at 5-year in time-dependent ROC curve analysis

The GSE39582 dataset included a total of 309 eligible CRC patients who were not treated with adjuvant chemotherapy, and these patients were enrolled in the analysis as the discovery cohort (Fig. 1A). Among 1531 FRGs from MSigDB v6.2, 1472 FRGs were measured on all platforms and thus were included in this study. After 1000 rounds of Cox univariate regression, 80% repeatable genes were chosen, and 76 FRGs were found to be related to patient DFS. After LASSO Cox regression analysis of stage II/III patients, 11 prognostic FRGs were ultimately confirmed and used to construct the FRGS (Fig. 1B). A satisfactory 5-year DFS cut-off identified via in time-dependent ROC curve analysis was used to train the FRGS for stratification of the high- and low-risk groups (Fig. 1C).

**Fig. 2:**
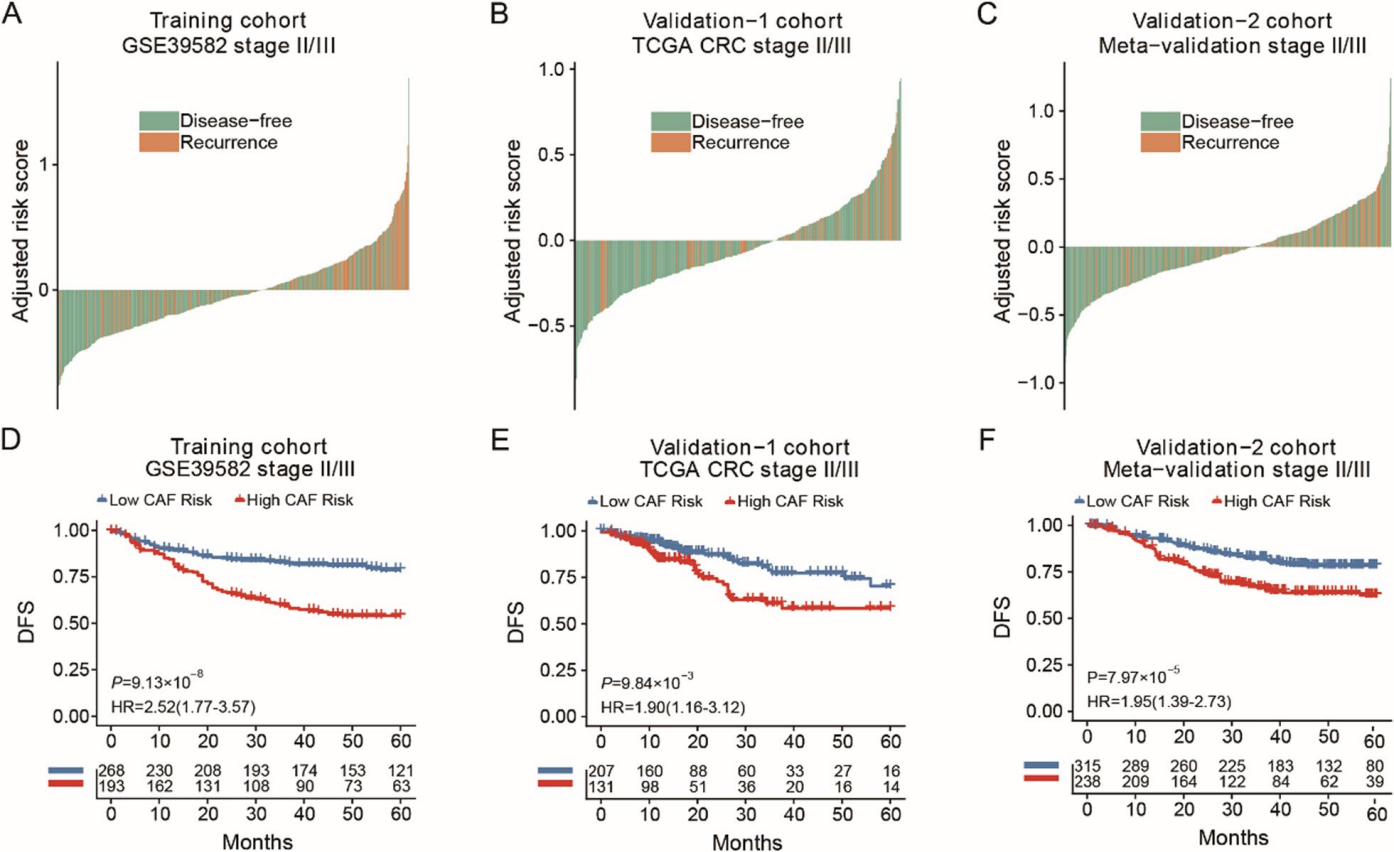
The outcome of different CAF risk in stage II/III CRC patients. **A**–**C** The DFS of patients with different CAF risk group in training cohort (**A**), TCGA cohort (**B**) and meta-validation cohort (**C**). **D**–**F** Kaplan–Meier curves comparing patients with different risk in training cohort (**D**), TCGA cohort (**E**) and meta-validation cohort (**F**)

The correlation coefficients between the genes in the FRGS and prognostic indexed were obtained (Table 1), and a risk score calculation model was used (Additional file 1: Fig. S1). Stage II/III CRC patients in the GSE39582 dataset (n = 461) were then used as the training cohort. More patients with tumour recurrence were found in the high-risk group than in the low-risk group in both the training and the two validation cohorts (Fig. 2A–C).

**Table 1:** The list of 11-Gene fibroblastic signatures

Gene	Function	Frequency in resampling	Average P-value	Coefficient
POLR2B	RNA polymerase II subunit B	1000	< 0.001	− 0.172
GAS6	Growth arrest specific 6	830	0.030	0.057
CRY1	cryptochrome circadian regulator 1	985	0.007	− 0.027
BCL2L1	BCL2 like 1	871	0.025	0.028
ARG1	Arginase 1	1000	0.001	0.075
ORAI3	ORAI calcium release-activated calcium modulator 3	999	0.003	0.010
TRAF3	TNF receptor associated factor 3	957	0.012	− 0.0004
ZSWIM4	Zinc finger SWIM-type containing 4	926	0.016	0.106
IRF1	Interferon regulatory factor 1	990	0.007	− 0.080
LEMD1	LEM domain containing 1	988	0.006	0.070
ACTB	Actin beta	906	0.021	0.044

### Validation of the FRGS in the TCGA and meta-validation cohorts

The other five CRC transcriptional datasets with prognostic data, including the TCGA dataset (n = 338) and the meta-validation cohort (n = 553) (combination of GSE17536, GSE33113, GSE37892, and GSE14333), were used as validation cohorts for the evaluation of the prognostic value of the FRGS. The values of the FRGS for patients in all stages were shown in Data S1 (Additional file 2: Data S1). No significant difference was observed among the three cohorts regarding the clinical and pathologic factors (date not shown).

Focusing on the stage II/III patients, the low- and high-risk groups stratified by the same risk score calculation model showed a significant difference in DFS in the training cohort (HR = 2.52, 95% CI 1.77–3.57, *P* < 0.001) (Fig. 2D), TCGA validation cohort (HR = 1.90, 95% CI 1.16–3.12, *P* < 0.01) (Fig. 2E) and meta-validation cohort (HR = 1.95, 95% CI 1.39–2.73, *P* < 0.001) (Fig. 2F). Furthermore, the FRGS showed satisfactory prognostic value in all CRC cohorts regardless of stage (CSE39582 cohort, HR = 1.91, 95% CI 1.41–2.58, *P* < 0.001; TCGA cohort, HR = 2.06, 95% CI 1.37–3.09, *P* < 0.001; meta-validation cohort, HR = 1.95, 95% CI 1.42–2.66, *P* < 0.001).

### The FRGS predicts benefit from chemotherapy in stage II/III CRC patients

Univariate and multivariate analyses were employed to investigate the association between sex, age, tumour location, tumour stage, gene aberration status, and FRGS score and CRC patient outcomes. In the univariate analysis, high risk identified by the FRGS was related to significantly worse prognosis in the training cohort (HR = 3.33, 95% CI 1.95–5.68, *P* < 0.001), TCGA cohort (HR = 5.00, 95% CI 1.58–15.85, *P* < 0.01) and meta-validation cohort (HR = 2.99, 95% CI 1.44–6.21, *P* < 0.01) (Table 2). Similarly, FRGS score remained an independent prognostic factor in the multivariate analysis (CSE39582 cohort, HR = 3.19, 95% CI 1.88–5.41, *P* < 0.001; TCGA cohort, HR = 5.00, 95% CI 1.58–15.85, *P* < 0.01; meta-validation cohort, HR = 2.99, 95% CI 1.44–6.21, *P* < 0.01) (Table 2).

**Table 2:** Univariate and multivariate analysis of FRGS, clinical and pathologic factors with DFS of stage II/III patients in training and validation cohorts

Characteristic	Training cohort (GSE39582, n = 461)				Validation-1 cohort (TCGA, n = 338)				Validation-2 cohort (meta-validation, n = 553)			
	Univariate		Multivariate		Univariate		Multivariate		Univariate		Multivariate	
	HR (95% CI)	*P* value	HR (95% CI)	*P* value	HR (95% CI)	*P* value	HR (95% CI)	*P* value	HR (95% CI)	*P* value	HR (95% CI)	*P* value
FRGS	3.33 (1.95–5.68)	< 0.001	3.19 (1.88–5.41)	< 0.001	5.00 (1.58–15.85)	< 0.01	5.00 (1.58–15.85)	< 0.01	3.25 (1.55–6.81)	< 0.01	2.99 (1.44–6.21)	< 0.01
Gender	1.53 (0.89–2.62)	0.12			1.56 (0.81–2.99)	0.18			0.70 (0.42–1.16)	0.16		
Age	1.01 (0.99–1.03)	0.58			1.01 (0.98–1.04)	0.37			0.98 (0.97–1.00)	0.07		
Tumor location	1.08 (0.64–1.84)	0.78			1.09 (0.59–2.04)	0.78			0.82 (0.40–1.66)	0.58		
TNM stage	7.89 (1.11–55.91)	0.01	7.46 (1.05–52.99)	0.04	1.83 (0.76–4.41)	0.17			3.59 (1.30–9.93)	< 0.01	3.39 (1.22–9.40)	0.02
MMR status	1.63 (0.70–3.82)	0.25			0.64 (0.34–1.24)	0.18			0.88 (0.38–2.04)	0.76		
CIMP status	0.95 (0.44–2.02)	0.89							0.91 (0.36–2.28)	0.84		
CIN status	1.69 (0.75–3.81)	0.2										
TP53 mutation	1.39 (0.78–2.48)	0.27							2.74 (0.60–12.43)	0.17		
KRAS mutation	1.44 (0.86–2.40)	0.16			1.02 (0.23–4.60)	0.98			1.23 (0.53–2.87)	0.63		
BRAF mutation	1.42 (0.57–3.58)	0.45			0.00 (0.00–Inf)	0.72			2.04 (0.81–5.10)	0.12		

**Fig. 3:**
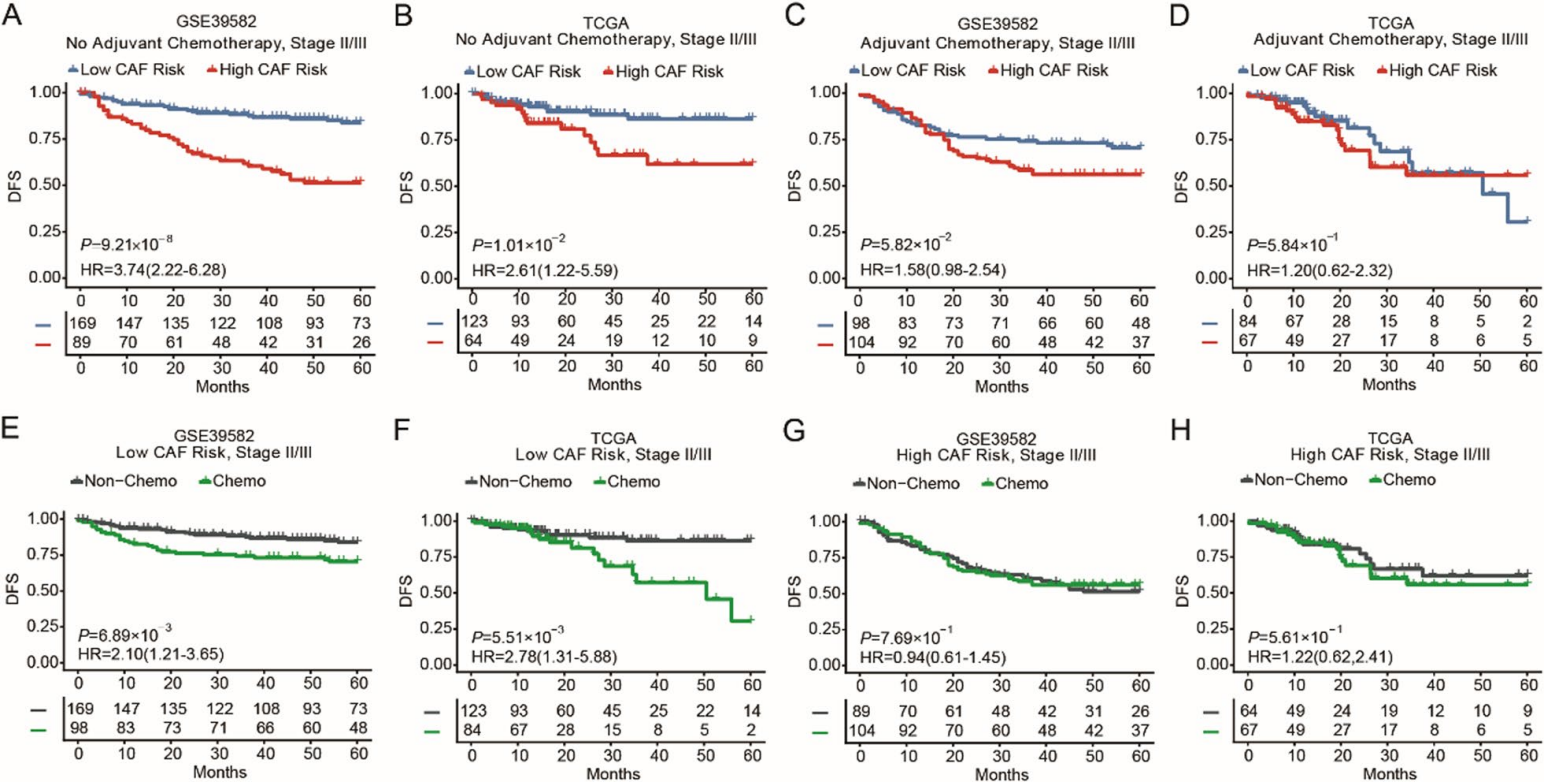
Kaplan–Meier plots for validations of FRGS in non-chemotherapy and chemotherapy cohorts. **A**, **B** The DFS of patients with different CAF risk group in stage II/III patients without adjuvant chemotherapy: GSE39582 cohort (**A**) and TCGA cohort (**B**). **C**, **D** The DFS of patients with different CAF risk group in stage II/III patients with adjuvant chemotherapy: GSE39582 cohort (**C**) and TCGA cohort (**D**). **E**, **F** The DFS of patients with or without adjuvant chemotherapy in stage II/III patients with low CAF risk: GSE39582 cohort (**E**) and TCGA cohort (**F**). **G**, **H** The DFS of patients with or without adjuvant chemotherapy in stage II/III patients with high CAF risk: GSE39582 cohort (**G**) and TCGA cohort (**H**)

To further clarify whether the FRGS could predict benefit from adjuvant chemotherapy in CRC patients, we focused on the clinical outcome in the nonchemotherapy and chemotherapy groups. In CRC patients who did not receive adjuvant chemotherapy, the DFS of high-risk group was worse than that of the low-risk group in both the training (HR = 3.74, 95% CI 2.22–6.28, *P* < 0.001, Fig. 3A) and TCGA cohorts (HR = 2.61, 95% CI 1.22–5.59, *P* = 0.01, Fig. 3B), but this pattern was not observed in patients treated with adjuvant chemotherapy (Fig. 3C, D, *P* > 0.05). Importantly, patients in the low-risk group were found to be benefit from chemotherapy (Fig. 3E, F, *P* < 0.01), but this was not seen in the high-risk group (Fig. 3G, H, *P* > 0.05).

### Functional assessment of the FRGS

**Fig. 4:**
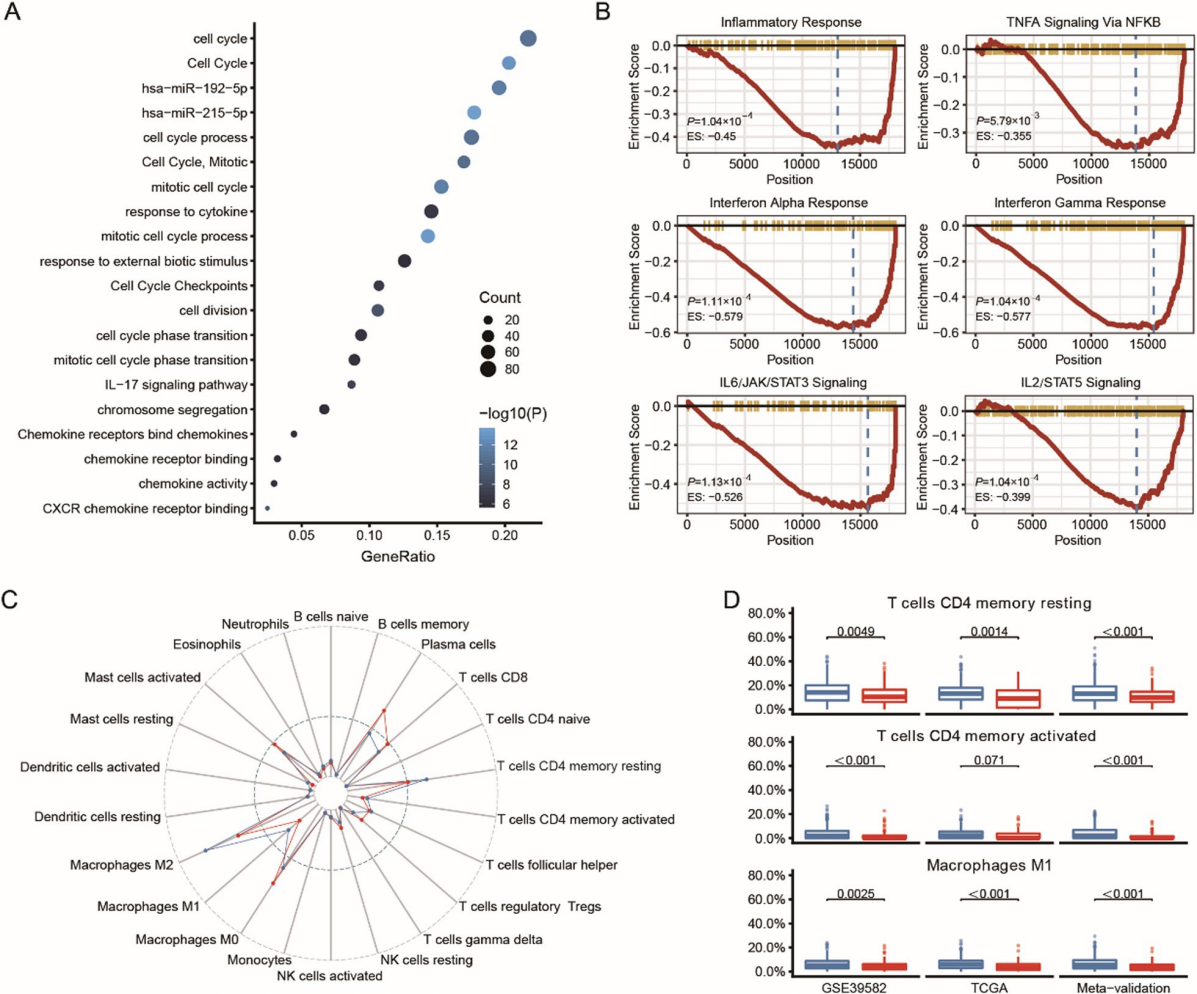
Functional annotation of FRGS. **A** Enrichment analysis of the differentially expressed genes between different groups in GO. **B** GSEA analysis showed inflammatory response, TNF-α, IFN-α, IFN-γ, IL-6 and IL-2 were depressed in high-risk patients. **C** Infiltrations of immune cells are assessed based on TCGA cohort. **D** CD4+ T cells and M1 macrophages were depressed in the high fibroblastic tumor

To explore the possible mechanism underlying the FRGS in high-risk patients, gene ontology (GO) enrichment analysis was performed to identify the biological processes of the differentially expressed genes. Interestingly, the most enriched biological processes other than the cell cycle were found to be associated with the immune response (Fig. 4A). To further evaluate the role of the FRGS, GSEA was performed in the TCGA CRC cohort, and the results showed that the CAF-induced microenvironment was significantly related to the inflammatory response, TNF-α, IFN-α, IFN-γ, IL-6 and IL-2 (Fig. 4B), which are related to the immune response network.

The ESTIMATE algorithm was further used to investigate the immune response in patients in different groups (Fig. 4C). Although no difference was found in the stromal score, immune score or ESTIMATE score between the two groups (data not shown), lower percentages of CD4+ T cells and M1 macrophages were detected in the FRGS-identified high-risk group (Fig. 4D).

### Assessment of the ability of the FRGS to predict the response to PD-1 treatment

**Fig. 5:**
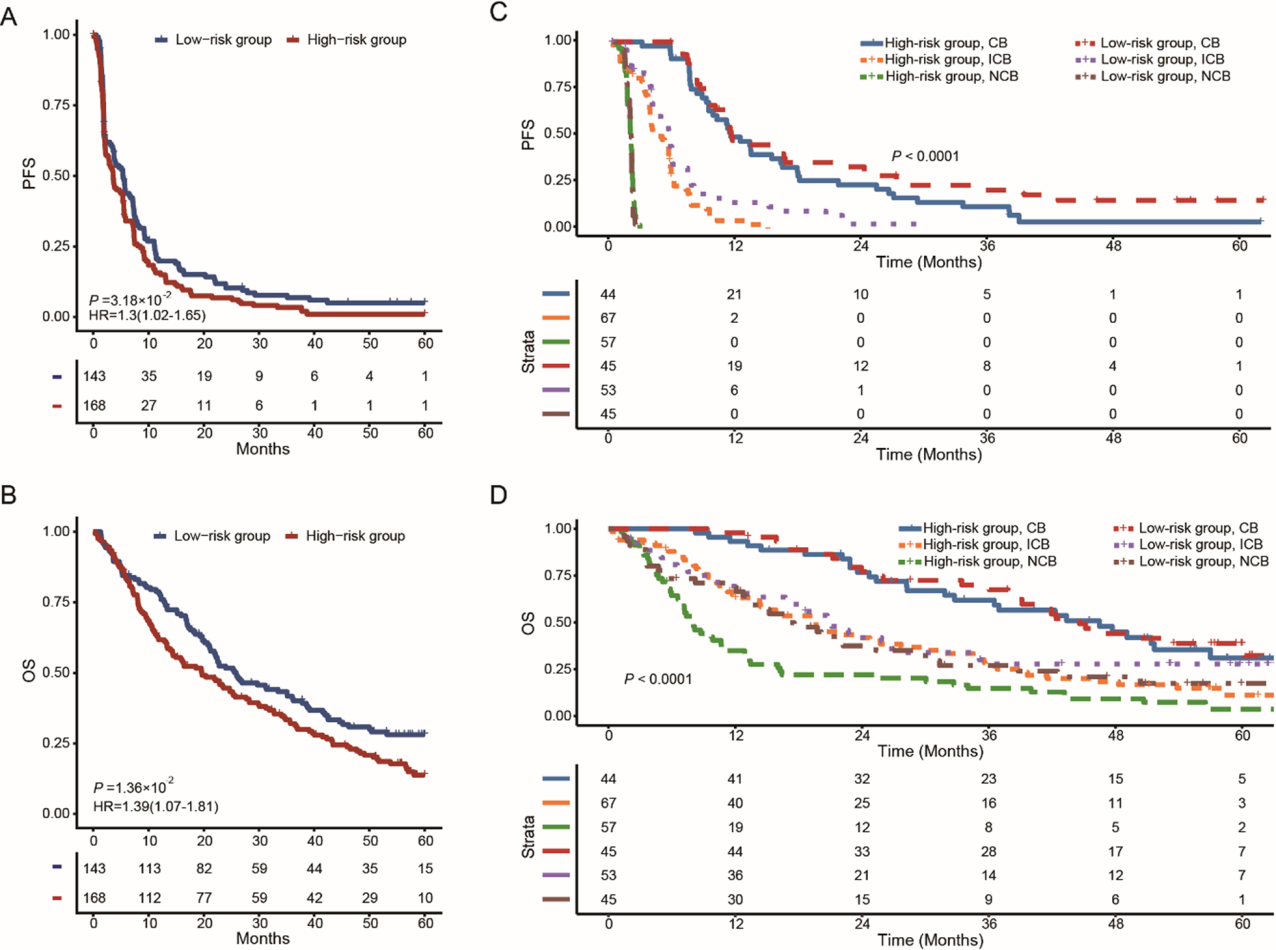
Kaplan–Meier plots for validations of FRGS in a cohort of ccRCC patients who received immunotherapy. **A**, **B** The outcome of patients with different CAF risk group in ccRCC patients with immunotherapy: PFS (**A**) and OS (**B**). **C**, **D** The outcome of patients with different drug response in ccRCC patients with different CAF risk group: PFS (**A**) and OS (**B**)

To evaluate whether the FRGS could be used to predict the response to immune checkpoint inhibitors, a cohort of advanced clear cell renal cell carcinoma (ccRCC) patients was used (Braun et al. 2020). With a cut-off value of − 0.026 (identified in the ROC curve analysis), patients were divided into a high-risk group and a low-risk group. The PFS and OS in the high-risk group were significantly shorter than those in the low-risk group (PFS: HR = 1.3, 95% CI 1.02–1.65, *P* = 0.0318, OS: HR = 1.39, 95% CI 1.07–1.81, *P* = 0.0136, Fig. 5A, B). In terms of immunotherapy benefit, patients in the low-risk group were found to have improved PFS (P < 0.0001) and OS (*P* < 0.0001) compared to those in the high-risk group. After stratification according to the drug response, clinical benefit (CB) and intermediate clinical benefit (ICB) patients in the FRGS-identified low-risk group had a longer DFS time (Fig. 5C), while no clinical benefit (NCB) patients in the FRGS-identified low-risk group had a longer OS time (Fig. 5D).

## Discussion

Based on our previous genome-wide analysis (Zou et al. 2019), we developed a fibroblast-related gene signature (FRGS) for the prediction of outcome in patients who have undergone resection for stage II/III CRC. A discovery cohort of stage II/III patients who did not receive adjuvant chemotherapy was first used to identify the genes, and the 11 fibroblast-related gene signature was then evaluated in multiple cohorts of stage II/III patients, including one training cohort and two independent validation cohorts. In these cohorts, patients in the high- and low-risk groups differed in HR for DFS by approximately 3 to 5 times in the univariate and multivariate analyses. Moreover, patients in the high-risk group did not benefit from chemotherapy. Some studies have indicated that chemotherapy-treated patients have a less than 5% improvement in the 5-year survival rate (Glynne-Jones et al. 2017; Lee et al. 2018), and the treatment might not reduce the risk of tumour recurrence (Draht et al. 2014; Gunderson et al. 2010; Nazemalhosseini Mojarad et al. 2016). Thus, patients in the high-risk group might be suggested to abandon adjuvant chemotherapy, as there is no improvement in survival, indicating that this new signature might be a useful supplement to the current TNM staging system and modify risk stratification for CRC patients.

Most of the genes in the FRGS have been previously found to be correlated with antitumour immune responses and tumour progression. In addition, Gas6 has been implicated in the promotion of tumour cell proliferation, survival, migration, invasion, angiogenesis, and immune evasion, which it accomplishes via Axl signalling (Tanaka and Siemann 2020). IRF1 has been identified as an important transcription factor for M1 macrophage polarization (Chu et al. 2021), which is consistent with our result that the infiltration of M1 macrophages was lower in the high-risk group (which had lower expression of IRF1). BCL2L1 (Bcl-xL) was found to be a driver in colorectal tumorigenesis and cancer progression (Scherr et al. 2016), which is in agreement with its protumour effect in the FRGS.

An increased proportion of fibroblasts in the tumour microenvironment has been reported to be correlated with poor prognosis in various cancer patients (Nishina et al. 2021; Song et al. 2021; Zhou et al. 2018). In CRC, a recent study showed that the expression of genes enriched in IL-11+ fibroblasts was correlated with reduced recurrence-free survival in patients, as IL-11+ fibroblasts activated both tumour cells and fibroblasts via secretion of IL-11; this phenomenon constituted a feed-forward loop between tumour cells and fibroblasts (Nishina et al. 2021). Although increasing laboratory evidence has revealed that CAFs are associated with tumorigenesis and chemoresistance, there is no tool that can distinguish high/low fibroblast-related risk and predict the prognostic response to chemotherapy in CRC, which hinders the use of tools related to fibroblasts in the clinic. In this study, we selected various FRGs to create a fibroblast-related gene signature (FRGS) for CRC patients. Our results indicated that the FRGS could stratify stage II/III CRC patients into different 5-year DFS rate groups, and the FRGS score also showed a better C-index than the prognostic tool Oncotype DX (Additional file 3: Table S1). These results indicated that the FRGS might be an effective prognostic tool because it can mirror the fibroblast status in CRC tumours.

To develop a novel therapeutic strategy against patients with high risk according to fibroblast-related parameters, it is important to understand the mechanism underlying fibroblast-related poor prognosis. To date, a mechanistic analysis of genome-wide data to determine how the interaction between CAFs and the tumour microenvironment induces a poor chemotherapy response is lacking. Many therapeutic methods may have effects on both tumour cells and CAFs. However, the underlying mechanisms by which chemotherapy affects the population and function of CAFs and how CAFs affect the treatment response remain unclear. Recent studies have indicated that chemotherapy can regulate the activation and function of CAFs. In addition, the cytokines and chemokines secreted by CAFs can also lead to chemoresistance by leading to degradation of extracellular matrix (ECM) and remodelling of blood vessels. Additionally, while tumours are exposed to a chemotherapy-induced toxic environment, the cross-talk between CAFs and tumour cells can also induce chemoresistance (Tang et al. 2018). In this study, functional analysis of the FRGS identified significantly lower immune cell infiltrations in the high-risk group. CAFs and immune cells act as major components and the most important cells in the TME. Recent studies have shown that CAFs are a major source of the immunosuppressive activity in the TME that may dictate the curative effect of immunotherapies (Barrett and Pure 2020). An early study indicated that CAFs contributed to the immunosuppressive phenotype based on a correlation between stromal markers and immunosuppressive cell types such as tumour-associated macrophages (TAMs) and myeloid-derived suppressor cells (MDSCs) (Zhou et al. 2018). CAFs can regulate TAMs by inducing LIF-mediated paracrine signalling, eliciting CCL2 expression for myeloid cell attraction to the TME, and silencing CXCL9 expression to inhibit the recruitment of cytotoxic T-cells (Pascual-Garcia et al. 2019). Most studies have shown the synergistic effect of CAFs and M2 macrophages on tumour progression, CAFs could also inhibit the proinflammatory features of M1 macrophages by reducing proinflammatory cytokine levels, nitric oxide production, migration, and the expression of M1 surface markers (Berzaghi et al. 2019). Patient-derived CAFs can induce the adhesion and enrichment of monocytes by upregulating the expression of VCAM1 and the secretion of IL-8 and promote the recruitment of M2 macrophages into the tumour microenvironment, which together inhibit the function of NK cells (Zhang et al. 2019). Additionally, CAFs also promoted the expression of immune checkpoints, such as TIM-3, PD-1, CTLA-4 and LAG-3, in CD4+ and CD8+ T-cells through the secretion of prostaglandin E2 (PGE2), contributing to a diminished immune function (Gorchs et al. 2019). In line with these data, we also found fewer CD4+ T cells and M1 macrophages in tumours with high levels of fibroblasts. These data suggest that the impact of fibroblasts on the tumour microenvironment is probably associated with immune suppression, which further enhances tumour progression. A microenvironment with high levels of fibroblast-related features could provide tumour cells with resistance to immunotherapy (Ai et al. 2015; Scharping et al. 2017), which has emerged as an effective therapy for CRC, and the FRGS may have the potential to predict the outcome of immunotherapy. Due to the lack of a CRC cohort that received immunotherapy, the FRGS was tested in a cohort of ccRCC patients and was found to have value in predicting the benefit of immunotherapy as well as clinical outcome. Further studies in CRC cohorts will be required to verify this hypothesis.

The limitations of this study are its retrospective design and the sample bias due to intratumour genetic heterogeneity (Mimori et al. 2018). Even though as many available datasets as possible were used to validate the FRGS, challenges might still exist owing to the lack of testing in a prospective cohort. Furthermore, the gene expression signature might be largely affected by sampling bias, and not all the batch effects could be addressed, although constant ordering and exclusion of FRGs were used to reduce the batch effects between different studies. Thus, the FRGS should be validated in a prospective cohort before its clinical application.

## Conclusion

In conclusion, the prognostic FRGS is a novel system that can be used to evaluate the DFS for stage II/III CRC patients and to stratify patients based on potential benefit from the adjuvant chemotherapy. CAFs in tumour microenvironment may promote chemoresistance in CRC patients by inhibiting the in situ antitumour immune response.

## Supplementary Information

**Additional file 1: Figure S1.** Risk score calculation model of FRGS

**Additional file 2:** **Data S1.** The risk of each patient in the six cohorts.

**Additional file 3:** **Table S1.** Comparation of FRGS and Oncotype in each dataset. 

## Data Availability

The datasets generated and analyzed during the current study are available in the GSE39582 (https://www.ncbi.nlm.nih.gov/geo/query/acc.cgi?acc=GSE39582), TCGA (https://www.cancer.gov/about-nci/organization/ccg/research/structural-genomics/tcga), GSE14333 (https://www.ncbi.nlm.nih.gov/geo/query/acc.cgi?acc=GSE14333), GSE17536 (https://www.ncbi.nlm.nih.gov/geo/query/acc.cgi?acc=GSE17536), GSE37892 (https://www.ncbi.nlm.nih.gov/geo/query/acc.cgi?acc=GSE37892) and GSE33113 (https://www.ncbi.nlm.nih.gov/geo/query/acc.cgi?acc=GSE33113).

## Abbreviations

CAFs: Cancer-associated fibroblasts
CB: Clinical benefit
ccRCC: Clear cell renal cell carcinoma
CRC: Colorectal cancer
DFS: Disease-free survival
ECM: Extracellular matrix
ESTIMATE: Estimation of STromal and Immune cells in MAlignant Tumor tissues using Expression data
FRGs: Fibroblast-related genes
FRGS: Fibroblast-related gene signature
GEO: Gene Expression Omnibus
GO: Gene ontology
GSEA: Gene set enrichment analysis
ICB: Intermediate clinical benefit
IQR: Interquartile ranges
LASSO: Least absolute shrinkage and selection operator
MDSCs: Myeloid-derived suppressor cells
NCB: No clinical benefit
OS: Overall survival
PFS: Progression-free survival
PGE2: Prostaglandin E2
ROC: Receiver operating characteristic
SD: Standard deviations
TAMs: Tumour-associated macrophages
TCGA: The Cancer Genome Atlas
TME: Tumour microenvironment
TPM: Transcripts per million
