# Supplementary figures and images for: Cancer-associated fibroblasts impact the clinical outcome and treatment response in colorectal cancer via immune system modulation: a comprehensive genome-wide analysis
Source: Mol Med. 2021 Oct 30;27:139. doi: 10.1186/s10020-021-00402-3 (PMC8557584; doi:10.1186/s10020-021-00402-3)

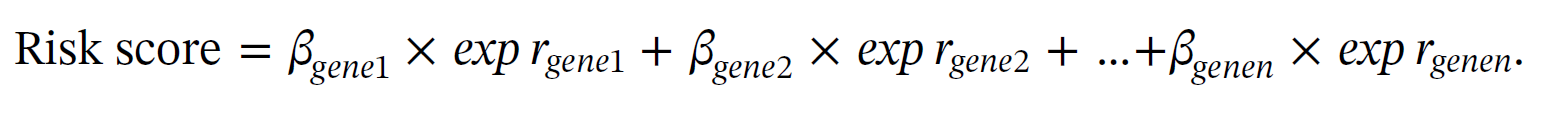


**Figure S1.** Risk score calculation model of FRGS

Supplement: Supplementary file 1 — Additional file 1: Figure S1. Risk score calculation model of FRGS [file 10020_2021_402_MOESM1_ESM.docx]
