# Supplementary material for: Cancer-associated fibroblasts impact the clinical outcome and treatment response in colorectal cancer via immune system modulation: a comprehensive genome-wide analysis
Source: Mol Med. 2021 Oct 30;27:139. doi: 10.1186/s10020-021-00402-3 (PMC8557584; doi:10.1186/s10020-021-00402-3)
Supplement: Supplementary file 3 — Additional file 3: Table S1. Comparation of FRGS and Oncotype in each dataset. [file 10020_2021_402_MOESM3_ESM.docx]

**Table S1.** Comparation of FRGS and Oncotype in each dataset

| Dataset | Clus | C.index |
| --- | --- | --- |
| GSE39582 | FRGS | 0.72 (0.62 - 0.83) |
|  | Oncotype DX colon | 0.65 (0.52 - 0.77) |
| TCGA | FRGS | 0.69 (0.53 - 0.84) |
|  | Oncotype DX colon | 0.61 (0.44 - 0.77) |
| GSE14333 | FRGS | 0.77 (0.59 - 0.96) |
|  | Oncotype DX colon | 0.64 (0.41 - 0.87) |
| GSE17536 | FRGS | 0.73 (0.49 - 0.96) |
|  | Oncotype DX colon | 0.72 (0.52 - 0.92) |
| GSE37892 | FRGS | 0.64 (0.29 - 1.00) |
|  | Oncotype DX colon | 0.59 (0.25 - 0.93) |
| GSE33113 | FRGS | 0.61 (0.39 - 0.83) |
|  | Oncotype DX colon | 0.82 (0.69 - 0.94) |
| GSE39084 | FRGS | 0.75 (0.48 - 1.00) |
|  | Oncotype DX colon | 0.43 (0.04 - 0.83) |
